# Prevalence and Associated Factors of Obesity among Panamanian Adults. 1982–2010

**DOI:** 10.1371/journal.pone.0091689

**Published:** 2014-03-12

**Authors:** Morris Sasson, Marcos Lee, Carmen Jan, Flavia Fontes, Jorge Motta

**Affiliations:** 1 Gorgas Memorial Institute for Health Studies, Panamá City, Panamá; 2 Department of Nutrition, Ministry of Health, Panamá City, Panamá; Johns Hopkins Bloomberg School of Public Health, United States of America

## Abstract

**Background:**

In Central America, there has been a marked increase in obesity in the last 30 years. Over this time frame, in Panama, there have been lifestyle changes associated with economic development and urbanization that may have facilitated increases in body weight. The aim of the study is to describe the change in the prevalence of obesity in the country since 1982 and to analyze the association of obesity with gender, place of residence and socioeconomic factors.

**Methods:**

We analyzed three nationally representative cross-sectional studies and one sub-national study of Panamanian adults that evaluated anthropometric and socioeconomic variables; ENPA-1982 (n = 11 611), ENV-II 2003 (n = 14 737), ENV-III 2008 (n = 15 484), PREFREC-2010 (n  = 3 590). We also evaluated one nationally representative study that evaluated people’s perception of their body weight, ENSCAVI-2007 (n = 25 748).

**Results:**

In 1982, the prevalence in males of a body mass index (BMI) ≥ 30 kg/m^2^ was 3.8% (3.3 – 4.2) and in females 7.6% (6.9 – 8.2). In 2003, the prevalence in males increased to 14.4% (13.6 – 15.2) and in females to 21.8% (20.8 – 22.7). In 2008, the prevalence in males was 16.9% (16.0 – 17.7) and in females it was 23.8% (22.8 – 24.7). Nevertheless, in 2007, the national perception of being obese was only 4% among males and 6.7% among females. The highest prevalence of obesity was noted in urban areas. Female gender and higher income were found to be positively associated with obesity. Income level was positively associated with abdominal obesity in men but not in women.

**Conclusions:**

There has been a marked increase of obesity in Panama in the last 3 decades. Initiatives to control this problem will have to take into consideration the observed gender difference and the lifestyle changes that have contributed to the rise of this problem.

## Introduction

Obesity is recognized as an important modifiable risk factor for ischemic heart disease, stroke, diabetes and cancer, diseases that are leading causes of morbidity and mortality. [1]–[5] Worldwide, mean BMI has increased for men and women and the prevalence rate of obesity has doubled in the past three decades. [6].

In developing countries, the prevalence of obesity has increased rapidly and in some it is as high or even higher than the prevalence reported in developed countries. [7], [8] Latin America has been one of the most affected regions of the world, with one of the largest rises in BMI occurring in Central and Mexico. [6].

In most countries, the prevalence of obesity is higher in women than in men, and higher in urban than in rural areas. [7] Females in Latin America have the third highest prevalence rate of obesity following the Middle East and Africa. [9].

Variables such as socioeconomic status (SES), ethnocultural background and place of residence have been said to play a role in the development of obesity.[3], [7], [10] Studies have shown a mixed association between obesity and SES, with a similar number of countries presenting positive and inverse associations or no association at all. [11], [12].

Most of Latin America has experienced rapid socioeconomic growth, accompanied by changes in nutrition characterized by increased consumption of energy-dense foods with elevated contents of fat and sugars. Another significant change has been an increase in urbanization, which has favored lifestyle modifications associated with decreased physical activity. [6], [13].

Panama is a country with a robust economic growth. The World Bank classifies Panama as country with an upper middle-income level. [14] Economic growth has accelerated the migration of people from rural to urban areas, which in turn has increased the number of people at risk of becoming overweight and obese because of changes in eating habits and daily physical activity leading to a more sedentary lifestyle. [4], [15]–[17].

The lifetime health and economic consequences of obesity are substantial, therefore many nations are dedicating public health efforts to prevent and reduce this important risk factor. [18], [19].

The purpose of this study is to describe changes in the prevalence of obesity in Panama since 1982, to assess its association to gender, place of residence and socioeconomic status, to identify main problematic areas and high-risk populations, and guide public health interventions.

## Materials and Methods

### Ethics Statement

Each study that we utilized as data source was approved by the Ethics Committee of the Ministry of Health of Panama and conducted in accordance with the declaration of Helsinki. All participants were informed about the objectives of the study and gave their written consent.

### Data sources

We searched for all published studies about obesity and overweight pertaining to Panama utilizing PubMed (National Library of Medicine, Bethesda, Maryland). We also reviewed unpublished reports and all national and sub-national surveys that measured BMI, including one study that evaluated self-reported estimation of body size. We obtained the data for analysis from the following studies (Table 1):

**Table 1:** Cross-sectional studies included in the present review.

Study	Year	Population study	Geographic area	Distribution of the population	Sampling method
ENPA	1982	11611 individuals W 54% M 46%	All provinces and indigenous areas	N/A	• Probabilistic
		≥18 years old			• Nationally representative
		Age mean: 36.1 SD:15.8			
ENV II	2003	14737 individuals W 51%, M 49%	All provinces and indigenous areas	Urban: 54%	• Multi-stage stratified
		≥18 years old		Rural: 39%	• Nationally representative
		Age mean: 40.3 SD: 16.5		Indigenous: 7%	
ENSCAVI	2007	25748 individuals W 60%, M 40%	All provinces and indigenous areas	Urban: 55%	• Multi-stage stratified,
		≥18 years old		Rural: 33%	• Randomized
		Age mean: 42.0 SD: 17.1		Indigenous: 12%	• Nationally representative
ENV III	2008	15484 individuals W 51%, M 49%	All provinces and indigenous areas	Urban: 67%	• Multi-stage stratified
		≥18 years old		Rural: 28%	• Nationally representative
		Age mean: 41.9 SD: 17.2		Indigenous: 5%	
PREFREC	2010	3590 individuals W 70%, M 30%	Panama and Colon provinces, 57.4% of the total population	Urban: 47%	• Stratified, randomized
		≥18 years old		Rural: 47%	• Sub-national representativeness
		Age mean: 45.4 SD: 16.4		Indigenous: 6%	

Abbreviations: ENPA, Estado Nutricional de la Población Adulta en Panamá; ENV, Encuesta de Niveles de Vida; ENSCAVI, Encuesta Nacional de Salud y Calidad de Vida; PREFREC, Prevalencia de Factores de Riesgo Asociados a Enfermedad Cardiovascular.

#### Estado Nutricional de la Población Adulta en Panamá (ENPA) – 1982

[20] This was the first nationally representative cross-sectional study that gathered anthropometric measurements from all the provinces of Panama. A sample of 8,299 households was randomly selected. From this sample, 11,611 individuals 18 years and older participated in the study and anthropometric measurements were obtained in all of them by trained health care workers.

#### Segunda Encuesta de Niveles de Vida (ENV) – 2003

[21] The ENV II of 2003 was a nationally representative, cross-sectional, observational study that used stratified multistage sampling. From a total of 26,435 households, 6,363 families were selected as the primary sampling unit. A total of 14,737 individuals 18 years and older participated in the study; anthropometric evaluations were performed on all of them by trained personal. A structured questionnaire was used to gather demographic, socioeconomic, health and anthropometric measurements. The ENV II study of 2003 was performed according to the Living Standard Measurements Study (LSMS) method, proposed by the World Bank. [22].

#### Tercera Encuesta de Niveles de Vida (ENV) – 2008

[23] The ENV III of 2008 was a nationally representative, cross-sectional, observational study that used stratified multistage sampling. From a total of 25,072 households, 8,000 families were selected as the primary sampling unit. A total of 15,484 individuals 18 years and older participated in the study and anthropometric measurements were obtained by trained personal. A structured questionnaire was used to gather demographic, socioeconomic, health and anthropometric measurements. The ENV III study of 2008 was performed according to the Living Standard Measurements Study (LSMS) method, proposed by the World Bank. [22].

#### Encuesta Nacional de Salud y Calidad de Vida (ENSCAVI) – 2007

[24] ENSCAVI - 2007 was a nationally representative cross-sectional population survey that utilized a multistage random sampling technique using a census population map to identify each district as the primary sampling unit. Out of 13,175 randomly selected dwellings, 25,748 subjects 18 years old and older were selected to participate in the study**.** Detailed interviews were performed using a validated questionnaire. The participants were asked about their self-perception of body size and about issues pertaining to their general health. Anthropometric measurements were not obtained in this study.

#### Prevalencia de Factores de Riesgo Asociados a Enfermedad Cardiovascular (PREFREC) – 2010

[25] PREFREC - 2010 was a sub-national, cross-sectional study done in 2010 in the two most populated provinces of the country, Panama and Colon. Approximately 57.4% of the total population of the country lives in these two provinces. Participants were selected using a stratified randomized sampling technique, from an estimated population of 1 009 326 subjects. The National Census of 2010 estimated that the population of Panama was 3,800,000. A total of 3590 participants 18 year of age participated in the study, and height, weight and abdominal circumference measurements were obtained by trained personal. A structured questionnaire was used to obtain information about ethnic, socioeconomic and health variables.

### Anthropometric variables

The BMI was calculated by dividing the weight in kilograms by the height in square meters (kg/m^2^) and categorized in three mutually exclusive groups based on the WHO and the Third Report of the National Cholesterol Education Program, Adult Treatment Panel III (NCEP-ATP III) criteria. Underweight was defined as a BMI of less than 18.5 kg/m^2^; normal weight was defined as BMI of 18.5–24.9 kg/m^2^; overweight was defined as BMI of 25–29.9 kg/m^2^; obesity was defined as a BMI ≥ 30 kg/m^2^. Abdominal obesity in women was defined as a waist circumference greater than 88 cm and in men more than 102 cm. [26].

### Socioeconomic variables

The national income levels that defined poverty or non-poverty status were utilized by the ENV of 2008. [23] In the ENV of 2008, extreme poverty referred to a yearly per capita income of ≤ $638 USD. Poverty was defined as a yearly per capita income of >$639<$1125 USD. Subjects with a yearly per capita income ≥ $1126 USD were defined as non-poor. [27] For the PREFREC study of 2010, monthly income levels were defined by strata, starting at an income of < $ 300 USD per month and ending at an income of > $1200 USD per month. Education levels were divided into basic education (pre–school, elementary, middle school), high school, and post–secondary (college/university and post-graduate school).

### Statistical analysis

Following identification and review, we extracted data pertaining to underweight, normal weight, overweight and obesity for all the provinces and indigenous regions. The prevalence of normal weight, overweight, obesity and mean BMI, with 95% confidence intervals (CI), was estimated for each province and for the country utilizing weights for the primary sampling units, for each province and for the nation. We included the ENPA 1982 estimates of obesity as the beginning of our analysis because there was no other national or sub-national information about obesity available for prior years.

An extended Mantel-Haenszel chi square test for linear trend was used to examine the linearity and significance of the changes in the prevalence of obesity from 1982 to 2008 and the changes in prevalence of obesity with regards to place of residence (urban, rural or indigenous areas) from 2003 to 2010. The obesity prevalence estimates were not age adjusted with a standard population.

For the ENV 2008 and PREFREC 2010 studies, logistic regression analyses were done to estimate the relationship between a BMI ≥30 kg/m^2^ and variables such as gender, place of residence, ethnocultural association, income level and education level. Results were expressed as odds ratios with 95% confidence intervals. All analyses were two-tailed and a P value <0.05 was considered statistically significant.

The statistical analyses were performed with SPSS Statistics version 20 (IBM, Dallas, Armonk, NY: IBM Corp USA) and with OpenEpi version 3.01, 2013 (http://www.openepi.com/). The thematic maps were made with the Manifold System Release 8.x Geographic Information System (GIS) package (Manifold Software Limited, Hong Kong).

## Results

**Figure 1:**
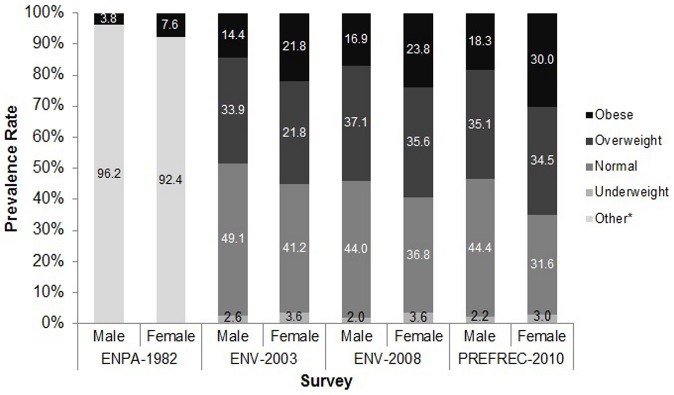
Prevalence of underweight, normal weight, overweight and obesity in Panamanian adults by gender and year of survey. Significant linear trend for prevalence estimates of obesity. Note: PREFEC-2010 is a sub-national two-province study.

The distribution of underweight, normal weight, overweight and obesity by gender and year of survey is presented in Figure 1. In the ENPA of 1982, the estimated national prevalence rate of obesity in adults was 3.8% (95% CI =  3.3 – 4.2) in males and 7.6% (95% CI =  6.9 – 8.2) in females. In the ENV II 2003 the estimated prevalence was 14.4% (95% CI =  13.6 – 15.2) in males and 21.8% (95% CI =  20.8 – 22.7) in females, and in the ENV III 2008, 16.9% (95% CI =  16.0 – 17.7) in males and 23.8% (95% CI =  22.8 – 24.7) in females. For both males and females, obesity prevalence changes followed a linear increase from 1982 to 2008 (p<0.0001). In the sub-national study PREFREC of 2010, which covered the provinces of Panama and Colon, the prevalence of obesity was 18.3% (95% CI =  16.5 – 20.0) in males and 30.0% (95% CI =  28.7 – 33.0) in females. Both, in the ENV III 2008 and in PREFREC 2010 there was a higher association of obesity with females than with males OR =  1.82 (1.68 1–.96, p< 0.001) and OR =  2.01 (CI =  1.68 – 2.4, p<0.001) respectively.

**Figure 2:**
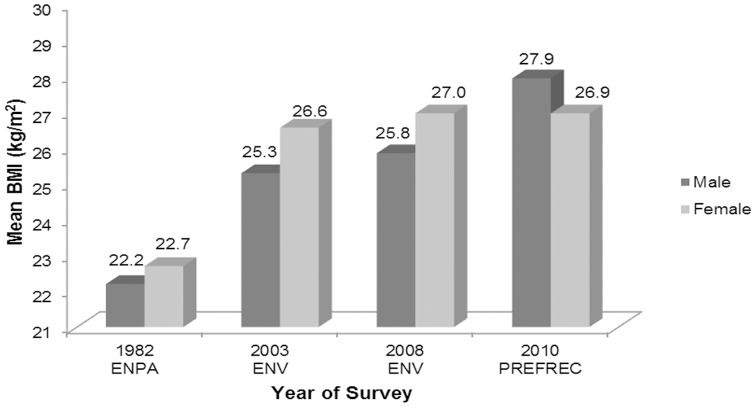
Mean BMI (kg/m^2^) in Panamanian adults by gender and year of survey. Note: PREFEC 2010 is a sub-national study.

The mean BMI by gender and year of survey are presented in Figure 2. In the ENPA 1982, the mean BMI for males was 22.2 kg/m^2^ (95% CI =  14.8 – 29.6) and 22.7 kg/m^2^ (95% CI =  13.9 – 31.5) for females; in the ENV II of 2003, the mean BMI for males was 25.28 kg/m^2^ (95% CI =  13.6 – 58.7) and 26.5 kg/m^2^ (95% CI =  13.1 – 56.5) for females; in the ENV III of 2008 the mean BMI for males was 25.84 kg/m^2^ (95% CI =  13.6 – 60.0) and for females 26.95 kg/m^2^ (95% CI =  11.9 – 57.4); in PREFREC 2010, the mean BMI for males was 27.92 kg/m^2^ (95% CI =  16.2 – 61.3) and for females 26.95 kg/m^2^ (95% CI =  13.5 – 62.8).

The prevalence of obesity by gender and by age groups (ENV 2003, ENV 2008, PREFREC 2010) is shown in Tables 2, 3 and 4. In all age groups, females had a greater prevalence of obesity than males. For females, the highest percentage of obesity was seen in the group aged 45 – 49 years except for ENV III study where the highest percentage was found in the 50 – 54 year old group. For males, the highest percentage of obesity was found in the in the 45 – 49 year old group in the three studies.

**Table 2:** Prevalence Rate of Obesity by Age Group of ENV 2003*.

Age Group	MALE(%)	CI 95%	FEMALE(%)	CI 95%	TOTAL(%)	CI 95%
18–24	5.8	4.3 – 7.3	11.5	10.0 – 13.0	8.7	7.7 – 9.8
25–29	11.6	9.6 – 13.7	18.7	16.9 – 20.6	15.2	13.9 – 16.6
30–34	13.8	11.6 – 16.0	20.4	18.4 – 22.3	17.3	15.8 – 18.7
35–39	18.5	16.0 – 20.9	23.8	21.8 – 25.8	21.3	19.8 – 22.9
40–44	18	15.5 – 20.4	29.1	26.9 – 31.2	23.8	22.1 – 25.4
45–49	21.3	18.7 – 23.9	33.2	30.9 – 35.4	27.5	25.8 – 29.2
50–54	15.7	13.4 – 18.0	29.9	27.8 – 32.1	22.7	21.1 – 24.3
55–59	15.2	12.9 – 17.4	29.2	27.0 – 31.3	22.5	20.9 – 24.1
60–64	15.7	13.4 – 18.0	27	24.9 – 29.1	21.2	19.7 – 22.8
65–69	14.6	12.3 – 16.8	26.3	24.2 – 28.4	20.4	18.9 – 21.9
70–74	9.7	7.8 – 11.5	27.4	25.2 – 29.5	18.6	17.1 – 20.1
75–79	8.6	6.8 – 10.4	15	13.3 – 16.7	11.6	10.3 – 12.8
≥80	3.5	2.4 – 4.7	9.6	8.2 – 11.0	6.9	5.9 – 7.8

Abbreviations: ENV 2003, Encuesta de Niveles de Vida 2003; CI, Confidence Intervals.

*National Survey; N = 14727.

**Table 3:** Prevalence Rate of Obesity by Age Group of ENV 2008*.

Age Group	MALE(%)	CI 95%	FEMALE(%)	CI 95%	TOTAL(%)	CI 95%
18–24	7.7	6.3 – 9.2	10.8	9.5 – 12.2	9.2	8.2 – 10.2
25–29	13.6	11.7 – 15.5	22	20.2 – 23.8	18	16.7 – 19.3
30–34	18.9	16.8 – 21.1	27.7	25.8 – 29.7	23.4	21.9 – 24.8
35–39	19.8	17.6 – 22.0	29.6	27.6 – 31.6	24.7	23.2 – 26.2
40–44	19.8	17.6 – 22.0	31	29.0 – 33.1	25.3	23.8 – 26.7
45–49	22.9	20.6 – 25.2	33.5	31.4 – 35.5	27.8	26.2 – 29.3
50–54	20.6	18.4 – 22.8	37.3	35.2 – 39.4	28.7	27.1 – 30.2
55–59	19.3	17.2 – 21.5	31.3	29.3 – 33.4	25.1	23.6 – 26.5
60–64	19.8	17.6 – 22.0	31.6	29.6 – 33.6	25.5	24.0 – 27.0
65–69	18.2	16.1 – 20.3	27.1	25.1 – 29.0	22	20.6 – 23.4
70–74	16	14.0 – 18.1	19.9	18.2– 21.7	18.2	16.9 – 19.5
75–79	11.9	10.1 – 13.7	17.1	15.5 – 18.7	15	13.8 – 16.3
≥80	3.8	2.7 – 4.8	14.1	12.6 – 15.6	9.3	8.3 – 10.3

Abbreviations: ENV 2008, Encuesta de Niveles de Vida 2003; CI, Confidence Intervals.

*National Survey; N = 15808.

**Table 4:** Prevalence Rate of Obesity by Age Group of PREFREC 2010*.

Age Group	MALE(%)	CI 95%	FEMALE(%)	CI 95%	TOTAL(%)	CI 95%
18–24	5.7	2.5 – 8.9	15.1	12.6 – 17.7	12.8	10.7 – 14.9
25–29	22.8	17.0 – 28.6	26.9	23.8 – 30.0	25.9	23.1 – 28.6
30–34	19.8	14.3 – 25.3	29.8	26.6 – 33.0	27.2	24.4 – 29.9
35–39	27.1	20.9 – 33.2	35.8	32.5 – 39.2	33.8	30.8 –36.7
40–44	23.4	17.5 – 29.2	34.8	31.5 – 38.1	31.6	28.7 – 34.5
45–49	23.6	17.7 – 29.5	42.2	38.7 – 45.6	37.3	34.3 – 40.3
50–54	21.9	16.2 – 27.6	35.4	32.1 – 38.8	31.5	28.6 – 34.4
55–59	23.3	17.5 – 29.1	35.1	31.7 – 38.4	31	28.1 – 33.9
60–64	11.5	7.1 – 15.9	35.4	32.0 – 38.7	27.1	24.3 – 29.9
65–69	16.5	11.4 – 21.6	37.3	33.9 – 40.7	28.9	26.1 – 31.7
70–74	19.2	13.7 – 24.6	25.7	22.7 – 28.8	23	20.4 – 25.7
75–79	10.4	6.2 – 14.6	16.7	14.1 – 19.3	13.9	11.7 – 16.0
≥80	9.1	5.1 – 13.1	9.5	7.5 – 11.6	9.3	7.5 – 11.1

Abbreviations: PREFREC 2010, Prevalencia de Factores de Riesgo Asociados a Enfermedad Cardiovascular; CI, Confidence Intervals.

*Subnational Survey: Panama and Colon; N = 3590.

**Figure 3:**
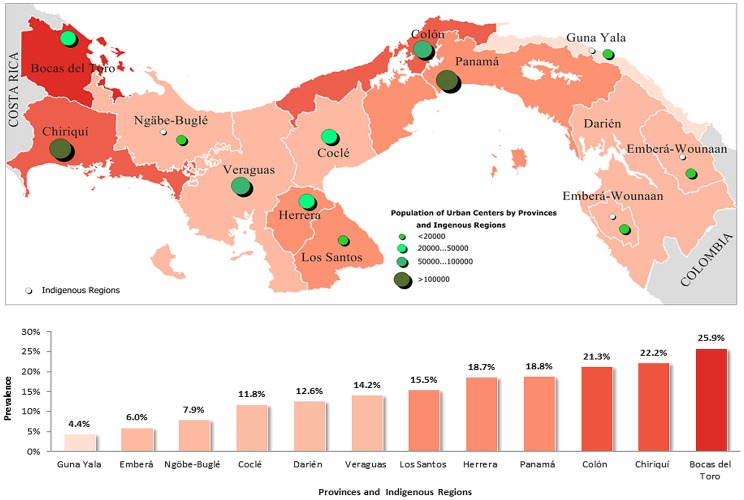
Mean prevalence rate of obesity in Panamanian adults by province and indigenous regions, according to the study ENV II - 2003. Population of urban centers and indigenous regions are identified.

**Figure 4:**
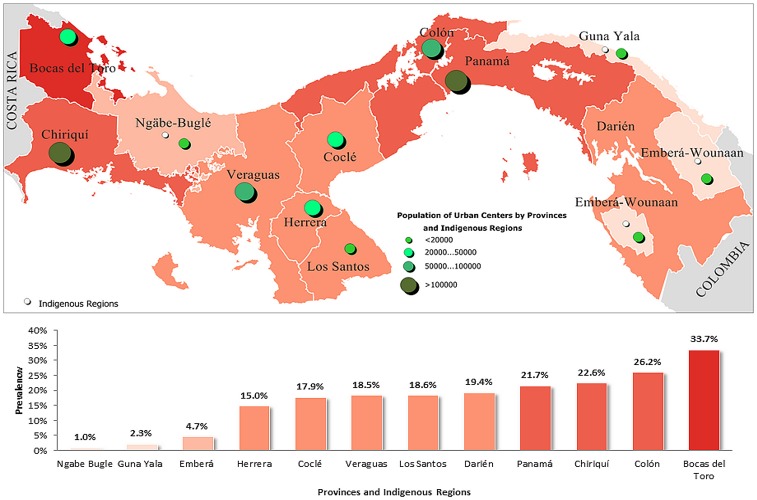
Mean prevalence rate of obesity in Panamanian adults by province and indigenous regions, according to the study ENV III - 2008. Population of urban centers and indigenous regions are identified.

Figures 3 and 4 represent the estimated prevalence rate of obesity in the provinces and indigenous regions of Panama according to the national surveys ENV II 2003 and ENV III 2008, respectively. In the ENV III 2008, the provinces of Bocas del Toro and Colon had the highest prevalence rate of obesity among adults, 33.7% and 26.2% respectively, while the indigenous regions had the lowest prevalence of obesity among adults, 2.3% in the Guna Yala region, 4.7% in the Emberá Wounaan region and 10.2% in the Ngäbe Buglé region.

**Figure 5:**
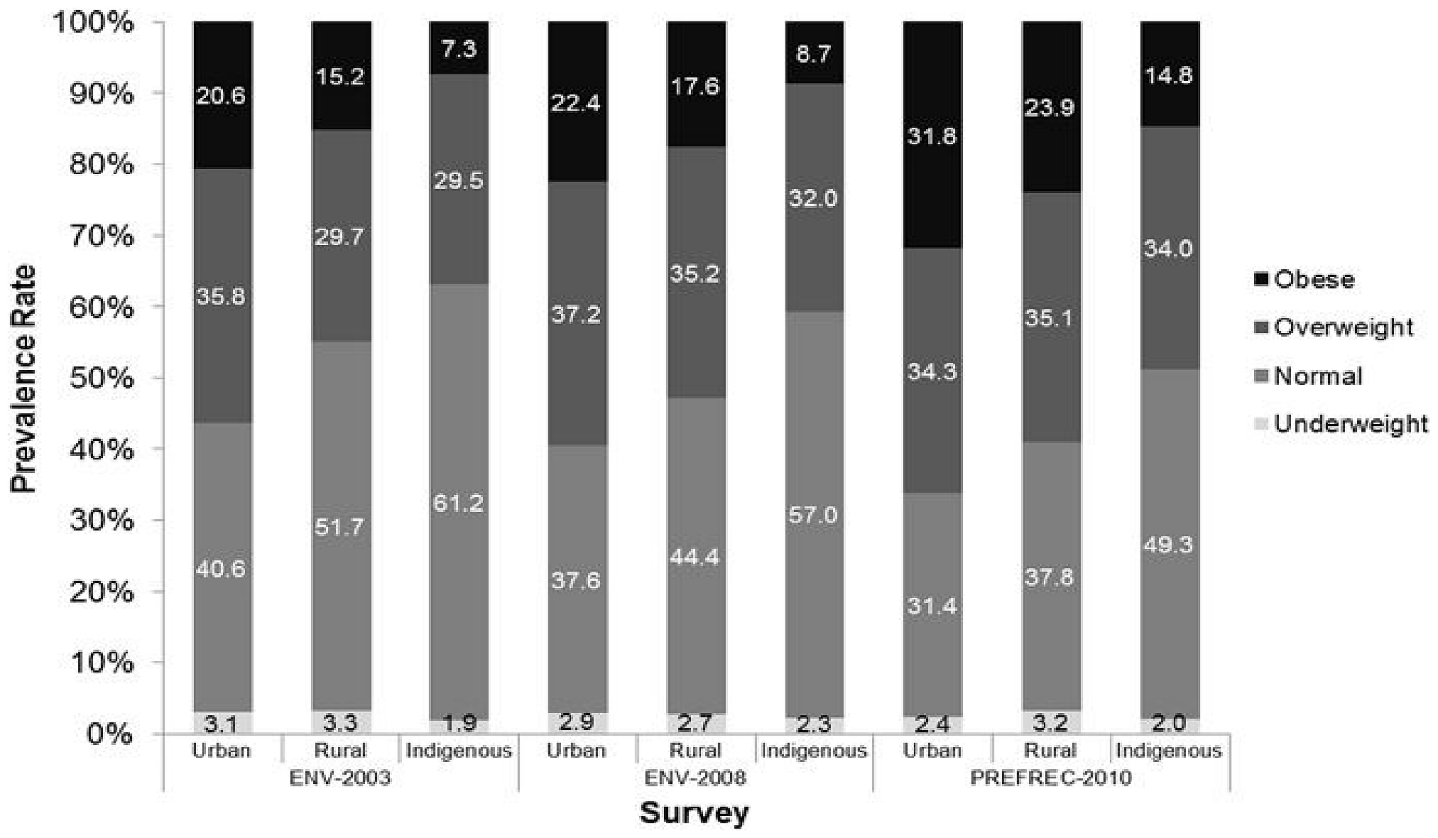
Prevalence of underweight, normal weight, overweight and obesity in Panamanian adults by geographic area (urban, rural and indigenous survey). Note: PREFEC-2010 is a sub-national study.

The distribution of underweight, normal weight, overweight and obesity by geographic area (urban, rural, indigenous regions), as estimated by the surveys ENV II 2003, ENV III 2008 and PREFREC – 2010, is presented in Figure 5, and in all areas it had an increasing trend (p<0.001). The ENV III 2008 showed that living in an urban area had a greater association with obesity than living in a rural area, OR = 1.24 (CI =  1.14 – 1.34, p< 0.001) or than living in an indigenous area, OR =  2.94 (CI = 2.41 – 3.58, p < 0.001). Similarly, PREFREC 2010 showed that living in an urban area had a greater association with obesity than living in a rural area, OR =  1.47 (CI = 1.26 – 1.71, p<0.001) or than living in an indigenous area, OR  =  2.71 (CI =  1.82 – 4.05, p<0.001).

**Figure 6:**
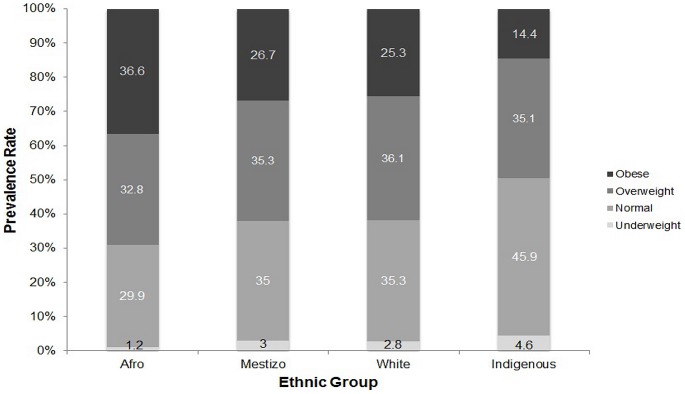
Prevalence of underweight, normal weight, overweight and obesity in Panamanian adults by ethnocultural identification in the study PREFREC of 2010.

Figure 6 presents the estimated prevalence of underweight, normal weight, overweight and obese among adults aged 18 and over by ethnocultural identification in the study PREFREC of 2010. Those who identified themselves as being of African origin had a greater risk of being obese when compared to those who described themselves as white OR =  1.71 (CI =  1.31 – 2.2, p<0.001), mestizo OR =  1.59 (CI = 1.33 – 1.90, p<0.001) or Amerindian OR =  3.47 (CI =  2.52 – 4.78 p<0.001). Furthermore, Amerindians living in urban areas had a lower percentage of obesity (17.0%) than those that identified themselves as white (27.0%), mestizos (31.4%) or of African origin (38.7%).

In ENV III 2008, those classified as non-poor or living above the poverty line (yearly per capita income ≥ $1126 USD) had a greater association with obesity OR = 2.34 (CI =  2.04 – 2.69, p< 0.001) when compared to those classified as extremely poor (yearly per capita income of ≤ $638 USD) or with those classified as poor OR = 1.66 (CI =  1.41–1.94) (yearly per capita income > $638 <$1126 USD). In PREFEC 2010, subjects with the highest monthly income levels were compared with those with the lowest income level (>$1200 USD vs ≤ $300 USD). In men, abdominal obesity was associated with the highest income levels, OR = 3.52 (CI =  1.93–5.87, p<0.05), while among women, there was an inverse association, OR = 0.44 (CI =  0.45–0.88, p<0.05).

We did not find a statically significant difference in the prevalence of obesity between those with a university and postgraduate education than those with high school education OR =  0.98 (CI =  0.87 – 1.10, p = 0.68) or those with less than a high school education OR = 1.10 (CI =  0.99 – 1.22, p = 0.08).

**Figure 7:**
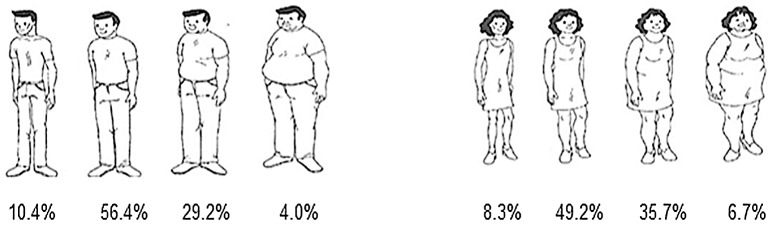
Self-reported perception of personal body complexion in Panamanian adults by gender, according to ENSCAVI - 2007 survey.

In the study ENSCAVI of 2007, participants were asked to evaluate their body weight using the images in Figure 7. Only 4% of males and 6.7% of females chose the profiles indicating the largest body size suggestive of obesity. By comparison, in the ENV III of 2008, anthropometric evaluation revealed that 16.9% of males and 23.8% of females had BMIs compatible with obesity.

## Discussion

There has been a marked increase in obesity in Panama for both males and females in the last 3 decades. The most recent nationally representative survey performed in 2008 estimated that approximately 56.4% of adults were either overweight or obese and that 21% were obese. Obesity in Panama is associated with being female, living in an urban area, being of African descent and being above the poverty line. These associations are similar to what has been described in studies performed in other countries. [17], [28]–[31].

While other South American countries, such as Colombia, Peru and Brazil, report lower prevalence rates, Uruguay reports a similar prevalence to the one estimated for Panama. [32], [33] Mexico and United States of America have a much higher prevalence of obesity than Panama and that of all other mentioned countries. [34], [35].

Studies employing proxy country data have estimated that Central America is one of the regions of the world where there has been one of the most rapid increases in the prevalence of obesity. [9], [36] Our results are in line with these estimates.

In Panama, obesity has increased in both genders, more among women, and also in urban, rural and indigenous areas. The ENV survey of 2008 estimated a mean BMI for women of 27 kg/m^2^ and of 25.8 kg/m^2^ for men. These estimates are consistent with the 2009 WHO estimate of a mean BMI of 27.9 kg/m^2^ for women and 26.4 kg/m^2^ for men. [37].

People who resided in indigenous areas had almost a 2 times lower prevalence of obesity than people who lived in the urban areas. We also found a lower prevalence of obesity among those who identified themselves as being Amerindians. This lower prevalence of obesity associated to this ethnocultural group may be, in part, due to lower socioeconomic status, diets with less calorie-dense foods and higher levels of physical activity during their daily activities. This relationship of a lower prevalence of obesity and indigenous status has also been identified in Mexico.[38], [39].

The association of being of African descent and obesity has been well documented in many countries. In the provinces of Panama and Colon, those who identified themselves as being of African origin had a greater association with obesity than whites, mestizos or Amerindians. Moreover, provinces with a high concentration of people of African descent like Bocas del Toro, predominantly rural or Colon, predominantly urban, had the highest prevalence of obesity in the nation. [28]–[30], [40], [41] This persistent association of obesity across rural and urban areas suggests a link of obesity with African ethno-cultural traits and behavior.

From 1980 to 2012, the per capita gross domestic product of Panama grew from US $ 1,915 to US $ 9534 [42] and the percentage of people living in urban areas grew from 50% to 75% or 2,500,000.[43] This rapid increase in economic growth and urbanization has resulted in nutritional changes linked to higher calorie consumption and lifestyle changes associated to reduced physical activity. [4], [15]–[17] Changes in occupation, transportation and technology directed at leisure time activities at home have also contributed to increased sedentary behavior. [6], [44].

From 2002 to 2011, the poverty rate in Panama fell from 48.5% to 27%, while extreme poverty decreased from 21% to 11%. [45] In our study we found that those classified as being above the poverty line had a greater association with obesity than those classified as being extremely poor. This unintended cost of economic prosperity probably comes about because, as people rise above the condition of extreme poverty and poverty, there is a greater likelihood of consumption of high-energy foods, especially inexpensive soft drinks, junk food and snacks that are ubiquitous in urban areas. [46], [47].

In men we found an association between higher income and abdominal obesity, but this was not the case for women. Similar results have been described in other countries. For women, a slender figure is socially valued and more often sought in higher income populations, while for men, larger body size is not a social handicap and may even be perceived as a sign of physical dominance. [31], [48]–[50].

We found that the perception of body size reported by the population studied in ENSCAVI 2007 was different than the national estimates for obesity determined by the ENV of 2008. This disparity raises our concern that while more than half of the adult population of the country is overweight or obese, the perception in the general population may be a different one. [51] Misperceptions of body image may act as a barrier for public health interventions focused at decreasing obesity prevalence. [52]–[54].

Numerous studies have shown that obesity increases morbidity and mortality and it has been identified as one leading preventable causes of disease in many parts of the world. [5], [55]–[58] The higher BMI and obesity rates documented in Panamanian women may be associated to the higher proportional mortality from diabetes seen in Panamanian women when compared to men. [59] Furthermore, the province of Colon, which has the second highest prevalence of obesity, has the highest age-adjusted rates of mortality for cerebrovascular disease and diabetes mellitus, and the second age-adjusted death rate for ischemic heart disease. [60], [61].

This study has the following limitations: The data from the ENPA of 1982 survey only allowed us to estimate the prevalence of the obese and the non-obese. Because of this shortcoming, we were not able to calculate the prevalence of overweight and normal weight, as we were able to do with the ENVII-2003 and the ENV III-2008. Nevertheless, these three nationally representative studies, designed in part to evaluate the nutritional state and weight of the Panamanian population, allow us to evaluate the changes of the prevalence of obesity that have occurred over the last twenty-five years.

The prevalence of obesity found in PREFEC cannot be used to estimate a trend of obesity in Panama since this study was sub-national. Furthermore, adding the results of PREFEC to a calculation of a national trend of obesity could bias the results because PREFREC was done in provinces with predominant urban development and with a higher prevalence of obesity.

We chose not to utilize age-adjusted summary statistics because we did not find a standard population that was suitable to age-adjust our 18-year and older population. Our age-specific obesity rates did not have a consistent relation with age, increasing progressively from young adulthood into middle age and decreasing as older age was reached. Moreover, our goal was to reflect the actual prevalence of obesity in the Panamanian population without losing information or deemphasizing some aspects of the data.

Potential selection bias, specifically sampling bias, may have occurred even though the sampling in all the studies included weights and stratification. In PREFREC more women than men were sampled and evaluated and this probably happened because more women than men were present at home the day of the survey.

Finally, the income distribution data available from the ENV 2008 and from PREFREC was not obtained as a continuous variable and the income distribution groupings was different in these two surveys. We were only able to include measurements of central obesity from the PREFREC 2010 study because waist circumference was not obtained in the ENPA and ENV surveys.

## Conclusions

Since 1982, there has been a marked increase in the prevalence of obesity in Panama. The prevalence of obesity has increased more in females and this may be associated to the higher rates of mortality from diabetes seen in Panama among females. Obesity was also associated to living in urban areas and being above the poverty line, characteristics of lifestyle and economic status that have rapidly changed over the last 3 decades in Panama. Government and private sector initiatives will have to be sensitive to factors like body weight perception, gender, and ethnocultural differences, and to the lifestyle changes that have triggered this rapid increase in obesity in Panama.

## References

[1] Chopra M, Galbraith S, Darnton-Hill I (2002) A global response to a global problem: the epidemic of overnutrition. Bulletin of the World Health Organization 80: 952–958. 12571723 PMC2567699

[2] MacDonald J, Brevard PB, Lee RE, Wagner T (2009) Link between diet and cardiovascular disease in Latin America and the Caribbean using geographic information systems. Revista panamericana de salud publica = Pan American journal of public health 26: 290–298. 20107676 10.1590/s1020-49892009001000002

[3] Garcia-Garcia E, De la Llata-Romero M, Kaufer-Horwitz M, Tusie-Luna MT, Calzada-Leon R, et al (2008) [Obesity and the metabolic syndrome as a public health problem: a reflection]. Salud Publica Mex 50: 530–547. 19039443 10.1590/s0036-36342008000600015

[4] Webber L, Kilpi F, Marsh T, Rtveladze K, Brown M, et al (2012) High rates of obesity and non-communicable diseases predicted across Latin America. PloS one 7: e39589. 22912663 10.1371/journal.pone.0039589 PMC3418261

[5] Calle EE, Rodriguez C, Walker-Thurmond K, Thun MJ (2003) Overweight, Obesity, and Mortality from Cancer in a Prospectively Studied Cohort of U.S. Adults. New England Journal of Medicine 348: 1625–1638. 12711737 10.1056/NEJMoa021423

[6] Alvarez V, Cuevas A, Olivos C (2009) The emerging obesity problem in Latin America. Expert Review of Cardiovascular Therapy 7: 281+. 10.1586/14779072.7.3.281 19296766

[7] Filozof C, Gonzalez C, Sereday M, Mazza C, Braguinsky J (2001) Obesity prevalence and trends in Latin-American countries. Obesity Reviews 2: 99–106. 12119667 10.1046/j.1467-789x.2001.00029.x

[8] WHO website. Obesity and Overweight Factsheet - 2013. Available: http://www.who.int/mediacentre/factsheets/fs311/en/. Accessed 2013 Dic 10.

[9] Finucane MM, Stevens GA, Cowan MJ, Danaei G, Lin JK, et al (2011) National, regional, and global trends in body-mass index since 1980: systematic analysis of health examination surveys and epidemiological studies with 960 country-years and 9·1 million participants. The Lancet 377: 557–567. 10.1016/S0140-6736(10)62037-5 PMC4472365 21295846

[10] Monteiro CA, Conde WL, Popkin BM (2001) Independent effects of income and education on the risk of obesity in the Brazilian adult population. The Journal of nutrition 131: 881S–886S. 11238779 10.1093/jn/131.3.881S

[11] Dinsa GD, Goryakin Y, Fumagalli E, Suhrcke M (2012) Obesity and socioeconomic status in developing countries: a systematic review. Obesity Reviews 13: 1067–1079. 22764734 10.1111/j.1467-789X.2012.01017.x PMC3798095

[12] Chang VW, Lauderdale DS (2005) Income disparities in body mass index and obesity in the United States, 1971–2002. Archives of Internal Medicine 165: 2122. 16217002 10.1001/archinte.165.18.2122

[13] Cuevas A, Alvarez V, Carrasco F (2011) Epidemic of metabolic syndrome in Latin America. Current opinion in endocrinology, diabetes, and obesity 18: 134–138. 10.1097/MED.0b013e3283449167 21358406

[14] The World Bank website. The World Bank-Panama. Available: http://www.worldbank.org/en/country/panama. Accessed 2013 Oct 20.

[15] Ford ES, Mokdad AH (2008) Epidemiology of obesity in the Western Hemisphere. The Journal of clinical endocrinology and metabolism 93: S1–8. 18987267 10.1210/jc.2008-1356

[16] Popkin BM, Doak CM (1998) The obesity epidemic is a worldwide phenomenon. Nutrition reviews 56: 106–114. 9584495 10.1111/j.1753-4887.1998.tb01722.x

[17] Neuman M, Kawachi I, Gortmaker S, Subramanian SV (2013) Urban-rural differences in BMI in low- and middle-income countries: the role of socioeconomic status. The American journal of clinical nutrition 97: 428–436. 23283503 10.3945/ajcn.112.045997 PMC3742298

[18] Hill JO, Peters JC (1998) Environmental contributions to the obesity epidemic. Science 280: 1371–1374. 9603719 10.1126/science.280.5368.1371

[19] Thompson D, Edelsberg J, Colditz GA, Bird AP, Oster G (1999) Lifetime health and economic consequences of obesity. Archives of Internal Medicine 159: 2177. 10527295 10.1001/archinte.159.18.2177

[20] de Bermudez O, Parillon C, Valverde V, de Pinto A (1984) [Weight and height in an adult Panamanian population]. Archivos latinoamericanos de nutricion 34: 605–614. 6545642

[21] The World Bank website. Encuesta de Niveles de Vida (ENV) – 2003. Available: http://econ.worldbank.org/WBSITE/EXTERNAL/EXTDEC/EXTRESEARCH/EXTLSMS/0,,contentMDK:21588642~menuPK:4196952~pagePK:64168445~piPK:64168309~theSitePK:3358997,00.html. Accessed 2013 Oct 21.

[22] The World Bank website. Living Standards Measurement Study. Available: http://go.worldbank.org/IPLXWMCNJ0. Accessed 2013 Apr 22.

[23] Contraloria Panama website. Encuesta de Niveles de Vida (ENV) – 2008. Available: http://www.contraloria.gob.pa/inec/Aplicaciones/ENV2008/intro.html. Accessed 2013 Oct 13.

[24] ICGES website. Encuesta Nacional de Salud y Calidad de Vida (ENSCAVI) – 2007. Available: http://www.gorgas.gob.pa/index.php?option=com_content&view=article&id=74&Itemid=143&lang=en. Accessed: 2013 Oct 21.

[25] ICGES website. Prevalencia de Factores de Riesgo Asociados a Enfermedad Cardiovascular (PREFREC) – 2010. Available: http://www.gorgas.gob.pa/index.php?option=com_content&view=article&id=73&Itemid=132&lang=en. Accessed 2013 Oct 21.

[26] National Cholesterol Education Program Expert Panel on Detection E and Treatment of High Blood Cholesterol in A (2002) Third Report of the National Cholesterol Education Program (NCEP) Expert Panel on Detection, Evaluation, and Treatment of High Blood Cholesterol in Adults (Adult Treatment Panel III) final report. Circulation 106: 3143–3421. 12485966

[27] The World Bank website. Panama - Encuesta de Niveles de Vida 2008. Available: http://microdata.worldbank.org/index.php/catalog/70 Accessed 2013 Oct 21.

[28] Cossrow N, Falkner B (2004) Race/ethnic issues in obesity and obesity-related comorbidities. The Journal of clinical endocrinology and metabolism 89: 2590–2594. 15181028 10.1210/jc.2004-0339

[29] Lovejoy JC, de la Bretonne JA, Klemperer M, Tulley R (1996) Abdominal fat distribution and metabolic risk factors: effects of race. Metabolism: clinical and experimental 45: 1119–1124. 8781299 10.1016/s0026-0495(96)90011-6

[30] Akil L, Ahmad HA (2011) Effects of socioeconomic factors on obesity rates in four southern states and Colorado. Ethnicity & disease 21: 58–62. 21462731 PMC3101796

[31] Xiao Y, Zhao N, Wang H, Zhang J, He Q, et al (2013) Association between socioeconomic status and obesity in a Chinese adult population. BMC public health 13: 355. 23590682 10.1186/1471-2458-13-355 PMC3656807

[32] Pisabarro R, Gutiérrez M, Bermúdez C, Prendez D, Recalde A, et al (2009) Segunda Encuesta Nacional de Sobrepeso y Obesidad (ENSO 2) adultos (18–65 años o más). Revista Médica del Uruguay 25: 14–26.

[33] Aballay LR, Eynard AR, Díaz MdP, Navarro A, Muñoz SE (2013) Overweight and obesity: a review of their relationship to metabolic syndrome, cardiovascular disease, and cancer in South America. Nutrition reviews 71: 168–179. 23452284 10.1111/j.1753-4887.2012.00533.x

[34] Rodriquez J RF, Peñaloza E, et al. Encuesta Nacional de Salud 2007. Resultados, Nacionales [in Spanish]. Bogotá CMdlP and 2009. S.

[35] Ogden CL, Carroll MD, Kit BK, Flegal KM (2012) Prevalence of obesity in the United States, 2009–2010. NCHS data brief: 1–8. 22617494

[36] Barcelo A, Gregg EW, Gerzoff RB, Wong R, Perez Flores E, et al (2012) Prevalence of diabetes and intermediate hyperglycemia among adults from the first multinational study of noncommunicable diseases in six Central American countries: the Central America Diabetes Initiative (CAMDI). Diabetes care 35: 738–740. 22323417 10.2337/dc11-1614 PMC3308278

[37] WHO website. Overweight/Obesity: Mean body mass index trends by country. Available: http://apps.who.int/gho/data/view.main.12461. Accessed 2013 Apr 23.

[38] Schulz LO, Bennett PH, Ravussin E, Kidd JR, Kidd KK, et al (2006) Effects of traditional and western environments on prevalence of type 2 diabetes in Pima Indians in Mexico and the U.S. Diabetes care. 29: 1866–1871. 10.2337/dc06-0138 16873794

[39] Stoddard P, Handley MA, Vargas Bustamante A, Schillinger D (2011) The influence of indigenous status and community indigenous composition on obesity and diabetes among Mexican adults. Social science & medicine 73: 1635–1643. 22033376 10.1016/j.socscimed.2011.09.006

[40] Clark AE, Taylor JY, Wu CY, Smith JA (2013) Alternative methods for measuring obesity in African American women. The Yale journal of biology and medicine 86: 29–39. 23483836 PMC3584493

[41] Liu J, Hickson DA, Musani SK, Talegawkar SA, Carithers TC, et al (2013) Dietary patterns, abdominal visceral adipose tissue, and cardiometabolic risk factors in African Americans: The Jackson heart study. Obesity (Silver Spring, Md) 21: 644–651. 10.1038/oby.2012.145 PMC3478414 23592674

[42] The World Bank website. GDP per capita. Available: http://data.worldbank.org/indicator/NY.GDP.PCAP.CD. Accessed: 2013 Oct 28.

[43] The World Bank website. Urban Population. Available: http://data.worldbank.org/indicator/SP.URB.TOTL.IN.ZS. Accessed 2013 Oct 25.

[44] Ebrahim S, Kinra S, Bowen L, Andersen E, Ben-Shlomo Y, et al (2010) The effect of rural-to-urban migration on obesity and diabetes in India: a cross-sectional study. PLoS medicine 7: e1000268. 20436961 10.1371/journal.pmed.1000268 PMC2860494

[45] The World Bank website. Panama Overview. Available: http://www.worldbank.org/en/country/panama/overview. Accessed 2013 Oct 27.

[46] Du S, Mroz TA, Zhai F, Popkin BM (2004) Rapid income growth adversely affects diet quality in China—particularly for the poor! Social science & medicine. 59: 1505–1515. 10.1016/j.socscimed.2004.01.021 15246178

[47] Uauy R, Albala C, Kain J (2001) Obesity trends in Latin America: transiting from under- to overweight. The Journal of nutrition 131: 893S–899S. 11238781 10.1093/jn/131.3.893S

[48] McLaren L (2007) Socioeconomic status and obesity. Epidemiologic reviews 29: 29–48. 17478442 10.1093/epirev/mxm001

[49] Dahly DL, Gordon-Larsen P, Popkin BM, Kaufman JS, Adair LS (2010) Associations between multiple indicators of socioeconomic status and obesity in young adult Filipinos vary by gender, urbanicity, and indicator used. The Journal of nutrition 140: 366–370. 20032487 10.3945/jn.109.114207 PMC2806889

[50] Seubsman SA, Lim LL, Banwell C, Sripaiboonkit N, Kelly M, et al (2010) Socioeconomic status, sex, and obesity in a large national cohort of 15-87-year-old open university students in Thailand. Journal of epidemiology/Japan Epidemiological Association 20: 13–20. 10.2188/jea.JE20090014 PMC3900775 19934589

[51] Loret de Mola C, Pillay TD, Diez-Canseco F, Gilman RH, Smeeth L, et al (2012) Body mass index and self-perception of overweight and obesity in rural, urban and rural-to-urban migrants: PERU MIGRANT study. PloS one 7: e50252. 23209688 10.1371/journal.pone.0050252 PMC3508895

[52] Herman KM, Hopman WM, Rosenberg MW (2013) Self-rated health and life satisfaction among Canadian adults: associations of perceived weight status versus BMI. Quality of life research : an international journal of quality of life aspects of treatment, care and rehabilitation. 10.1007/s11136-013-0394-9 23539466

[53] Kuchler F, Variyam JN (2003) Mistakes were made: misperception as a barrier to reducing overweight. International journal of obesity and related metabolic disorders : journal of the International Association for the Study of Obesity 27: 856–861. 10.1038/sj.ijo.0802293 12821973

[54] Lemon SC, Rosal MC, Zapka J, Borg A, Andersen V (2009) Contributions of weight perceptions to weight loss attempts: differences by body mass index and gender. Body image 6: 90–96. 19188102 10.1016/j.bodyim.2008.11.004 PMC2692706

[55] Adams KF, Schatzkin A, Harris TB, Kipnis V, Mouw T, et al (2006) Overweight, Obesity, and Mortality in a Large Prospective Cohort of Persons 50 to 71 Years Old. New England Journal of Medicine 355: 763–778. 16926275 10.1056/NEJMoa055643

[56] Gunnell DJ, Frankel SJ, Nanchahal K, Peters TJ, Davey Smith G (1998) Childhood obesity and adult cardiovascular mortality: a 57-y follow-up study based on the Boyd Orr cohort. The American journal of clinical nutrition 67: 1111–1118. 9625081 10.1093/ajcn/67.6.1111

[57] Mokdad Ah SMKDWHBBAMJSKJP (2000) THe continuing epidemic of obesity in the united states. JAMA 284: 1650–1651. 11015792 10.1001/jama.284.13.1650

[58] Solomon CG, Manson JE (1997) Obesity and mortality: a review of the epidemiologic data. The American journal of clinical nutrition 66: 1044S–1050S. 9322585 10.1093/ajcn/66.4.1044S

[59] Motta JA, Ortega-Paz LG, Gordon CA, Gomez B, Castillo E, et al (2013) Diabetes mortality in Panama and related biological and socioeconomic risk factors. Revista panamericana de salud publica = Pan American journal of public health 34: 114–120. 24096976

[60] Motta J, Gordon C, Herrera V, Castillo E, Ortega L, et al. (2012) Análisis de la Mortalidad Asociada a Enfermedades circulatorias y Diabetes Mellitus en Panamá, 2001– 2011. Available: http://www.researchgate.net/publication/233920133_Analisis_de_la_mortalidad_producida_por_enfermedades_cardiovasculares_y_diabetes_en_Panama. Accessed 2014 Jan 10.

[61] ICGES website. Tasa ajustada de mortalidad para todas las enfermedades circulatorias en Panamá según provincias. Available: http://www.gorgas.gob.pa/SiGCARDIOVASCULARES/Datos.htm. Accessed: 2014 Jan 10.

